# Establishment of isotype-switched, antigen-specific B cells in multiple mucosal tissues using non-mucosal immunization

**DOI:** 10.1038/s41541-023-00677-z

**Published:** 2023-05-31

**Authors:** John T. Prior, Vanessa M. Limbert, Rebecca M. Horowitz, Shaina J. D’Souza, Louay Bachnak, Matthew S. Godwin, David L. Bauer, Jaikin E. Harrell, Lisa A. Morici, Justin J. Taylor, James B. McLachlan

**Affiliations:** 1 Department of Microbiology and Immunology, Tulane University School of Medicine, New Orleans, Louisiana USA; 2 Vaccine and Infectious Disease Division, Fred Hutchinson Cancer Research Center, Seattle, WA USA; 3 Department of Global Health, University of Washington, Seattle, WA USA; 4 Department of Immunology, University of Washington, Seattle, WA USA

**Keywords:** Adjuvants, Germinal centres, Class switch recombination, Protein vaccines, Antibodies

## Abstract

Although most pathogens infect the human body via mucosal surfaces, very few injectable vaccines can specifically target immune cells to these tissues where their effector functions would be most desirable. We have previously shown that certain adjuvants can program vaccine-specific helper T cells to migrate to the gut, even when the vaccine is delivered non-mucosally. It is not known whether this is true for antigen-specific B cell responses. Here we show that a single intradermal vaccination with the adjuvant double mutant heat-labile toxin (dmLT) induces a robust endogenous, vaccine-specific, isotype-switched B cell response. When the vaccine was intradermally boosted, we detected non-circulating vaccine-specific B cell responses in the lamina propria of the large intestines, Peyer’s patches, and lungs. When compared to the TLR9 ligand adjuvant CpG, only dmLT was able to drive the establishment of isotype-switched resident B cells in these mucosal tissues, even when the dmLT-adjuvanted vaccine was administered non-mucosally. Further, we found that the transcription factor Batf3 was important for the full germinal center reaction, isotype switching, and Peyer’s patch migration of these B cells. Collectively, these data indicate that specific adjuvants can promote mucosal homing and the establishment of activated, antigen-specific B cells in mucosal tissues, even when these adjuvants are delivered by a non-mucosal route. These findings could fundamentally change the way future vaccines are formulated and delivered.

## Introduction

One of the most important observations in vaccinology over the last decade, and the one with the most potential to transform the way vaccines are designed and delivered, is the observation that mucosal immune responses can be generated by vaccinating via non-mucosal (parenteral) routes, especially intradermally^1–3^. Most currently licensed vaccines are delivered by injection, predominantly intramuscularly. While excellent at inducing systemic immunity, the mucosal immune response to these vaccines is often limited at best. These vaccines are designed to induce high systemic anti-pathogen antibody titers that target the invading pathogen for elimination. This antibody production is dependent on the initial activation of naïve vaccine-specific B cells that subsequently proliferate and begin to produce vaccine-specific antibodies^4^. While some activated B cells differentiate into short-lived plasma cells and immediately secrete predominantly lower affinity antibodies^5^, other vaccine-specific B cells can undergo maturation to become long-lived plasma cells that eventually migrate to the bone marrow and produce anti-pathogen antibodies for months or even decades^6^. Successful vaccines also induce long-lived memory B cells, which can quickly respond to re-infection and often secrete higher affinity antibodies due to somatic hypermutation during the initial exposure to the vaccine. Notably, re-exposure to pathogens or a vaccine can further increase antibody affinity, which is why most vaccines are delivered as two- or even three-dose regimens^7,8^.

While some vaccines, such as the current measles, mumps, and rubella vaccine, are comprised of live attenuated pathogens, it is becoming more common, for safety reasons, that vaccines are composed of pieces, or “subunits” of pathogens. While live attenuated vaccines are often highly immunogenic due to their replicating nature and expression of multiple danger signals, subunit vaccines typically require the inclusion of an immunomodulating agent, called an adjuvant, that can help elicit a more robust B or T cell response^9^. Currently, the few adjuvants utilized in injectable vaccines, including aluminum salts (alum) alone, alum combined with monophosphoryl lipid A (MPL-A) as the adjuvant AS04, and CpG oligodeoxynucleotide (CpG), excel at inducing an effective systemic IgG response but generally lack the ability to induce mucosal immunity^10–12^. It is becoming appreciated that injectable vaccination must be combined with an appropriate adjuvant to achieve mucosal immunity. Only a small number of injected adjuvants can promote this mucosal immunity, including bacterial ADP-ribosylating toxin adjuvants such as cholera toxin; however, most do not. We recently showed that the non-toxic, ADP-ribosylating adjuvant double mutant heat-labile toxin (dmLT) can drive vaccine-specific CD4 T cells into the gut mucosa^13^, as well as elicit vaccine-specific lung CD4 T cells^14^. The lack of understanding of how the route and adjuvant choice affect vaccine-induced mucosal immunity has hampered the field^1,2,13,15–17^. While most studies using ADP-ribosylating adjuvants have assessed mucosal secretory antibodies such as IgA, few have explored whether non-mucosal immunization can induce migration of vaccine-specific B cells directly into mucosal tissues^2,15,16^. Indeed, no studies we are aware of have directly assessed the impact of ADP-ribosylating adjuvants on the activation and homing of vaccine-specific B cells themselves. This is important as recent work has identified a population of B cells that, like some T cells, take up residence in mucosal tissues^18,19^. These local mucosal B cells have been shown to play a significant protective role against a variety of pathogens, such as influenza in the lung or herpes simplex virus 2 in the vaginal lumen^18,20^. B cells that reside at mucosal surfaces are also able to respond rapidly to re-exposure and can subsequently deliver IgA directly into the lumen of mucosal tissues such as the gut and lung^18,21,22^. This local mucosal antibody response is likely more effective at clearing pathogens as IgA in the blood is rapidly eliminated from the body and less likely to prevent mucosal infection^18,23^. The importance of inducing a strong mucosal B cell response during vaccination is further exemplified by differences between the orally administered Sabin polio vaccine and the intramuscularly injected Salk polio vaccine. While both vaccines have contributed substantially to the decrease in polio infections, the parenterally administered Salk vaccine is unable to induce a vigorous secretory IgA response in the intestinal lumen, which allows infected people to continue to transmit poliovirus to other, uninfected individuals^24^. In contrast, the orally delivered Sabin vaccine prevents polio person-to-person transmission due to a secretory IgA response^25–27^.

To our knowledge, the adjuvant effect on endogenous, antigen-specific B cell activation and mucosal migration after parenteral vaccination has not been examined. Here we assessed how adjuvant choice regulates B cell activation and mucosal migration in response to vaccination using antigen tetramers that identify and phenotype endogenous, vaccine-specific B cells^28,29^. We show that a single intradermal vaccination, using dmLT as the adjuvant, induced a vigorous expansion of antigen-specific B cells that were isotype-switched and displayed a germinal center phenotype. While we were unable to locate these B cells in the mucosa following this single immunization, boosting the response drove isotype-switched vaccine-specific B cells into the gut and lungs, contrasting with the TLR9 agonist adjuvant CpG, which was unable to induce this migration_._ Importantly, these cells were not in the blood but, instead, were located in the mucosal tissues themselves. In addition, intradermal administration of dmLT was able to induce fecal IgA, while CpG was not. These results demonstrate, for the first time, that a non-mucosal vaccination strategy using the appropriate adjuvant can drive endogenous vaccine-specific B cells into a variety of mucosal tissues where they are likely to serve protective functions against pathogenic challenges. This has implications for future vaccines that could be designed to elicit mucosal B cell immunity via non-mucosal routes, better positioning us to combat the next pandemic mucosal infection such as that caused by SARS-CoV-2.

## Results

### dmLT induces a potent antigen-specific, isotype-switched, germinal center B cell response after a single injection

**Fig. 1:**
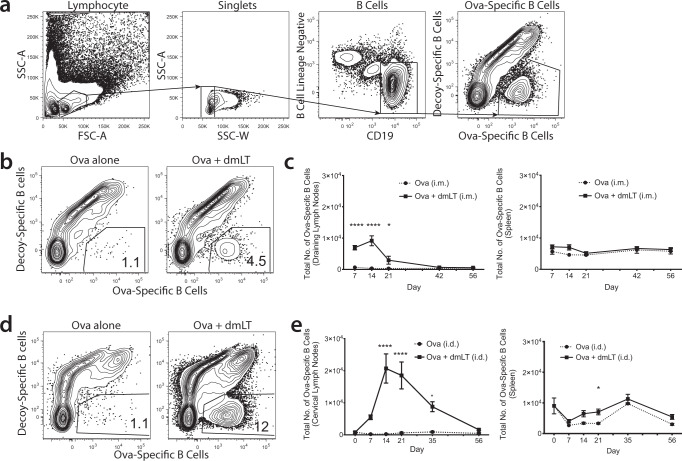
dmLT induces the greatest number of Ova-specific B cells at 14 days post-injection in the draining lymph nodes. **a** Gating strategy with representative flow plots. The B cell lineage negative gate was comprised of anti CD3ε, CD11c, and F4/80 antibodies. **b**, **c** Thighs of WT C57Bl/6 mice were intramuscularly injected with 10 μg of Ova or 10 μg of Ova plus 1 μg of dmLT. Mice were euthanized at the designated time points described in “Methods”. At each time point, inguinal, periaortic, and popliteal lymph nodes (combined as the draining lymph nodes (DLNs)) and the spleen were harvested and stained with decoy and tetramer. **b** Representative flow plots of the DLNs 14 days post intramuscular injection. **c** Total number of Ova-specific B cells during the time course is shown. **d**, **e** Ears of WT C57Bl/6 mice were intradermally injected with 10 μg of Ova or 10 μg of Ova plus 1 μg of dmLT. At each time point, cervical lymph nodes (CLNs) and the spleen were harvested and stained with decoy and tetramer. **d** Representative flow plots of the CLNs 14 days post intradermal injection. **e** Total number of Ova-specific B cells during the time course is shown. *p < 0.05; ****p < 0.0001. Statistical analysis was performed using a two-way ANOVA with Sidak’s multiple comparison test. The results shown are representative of two independent experiments, *N* = 5. Graphs represent the mean at each time point ± the S.E.M.

Since most vaccines are administered intramuscularly (IM), we assessed how a single IM immunization with dmLT as the adjuvant might affect endogenous antigen-specific B cell responses. Mice were injected IM with the antigen chicken egg ovalbumin (Ova) alone or in combination with dmLT. After 7, 14, 21, 42, and 56 days, we harvested injection-site draining lymph nodes (inguinal, periaortic, and popliteal) and spleens and assessed endogenous Ova-specific B cell numbers using an Ova tetramer specifically capable of binding to and identifying Ova-specific B cells. To control for, and eliminate, B cells specific for the non-Ova components of the tetramer, a decoy tetramer was employed (see “Methods” for the description of the tetramers). Using the gating strategy shown (Fig. 1a), Ova-specific B cells in mice receiving Ova alone remained low throughout the time course. In the draining lymph nodes in mice that received Ova plus dmLT, there was an increase in the number of Ova-specific B cells after IM injection peaking 2 weeks after injection and eventually returning to baseline by week 8 (Fig. 1b, c). There was no difference between Ova and Ova plus dmLT in the spleen. It is known that the route of injection can alter the immune response to vaccination^30,31^, and it has been shown that different infection routes can dramatically alter the phenotype of T cells depending solely on the infection route^32^. We previously demonstrated that a single intradermal (ID) injection of dmLT plus a model antigen drives a potent vaccine-specific CD4 helper T cell response in the draining lymph nodes^13^. We hypothesized that this same ID immunization would concurrently induce the expansion of antigen-specific B cells. To test this, mice were injected once ID with Ova, with or without dmLT. After 7, 14, 21, 35, and 56 days, the draining cervical lymph nodes (CLNs) and spleen were harvested. Mice that received Ova alone had a small increase of Ova-specific B cells that peaked 5 weeks after injection in the draining CLNs and spleen (Fig. 1d, e). In contrast, mice injected with Ova and dmLT showed a significant expansion of Ova-specific B cells starting on day 7, peaking on day 14, and returning to baseline by day 56 (Fig. 1e) in the CLNs. There was no difference in the number of Ova-specific B cells in the spleen when Ova and Ova plus dmLT were compared (Fig. 1e). Notably, this B cell expansion was higher overall compared to IM immunization.

**Fig. 2:**
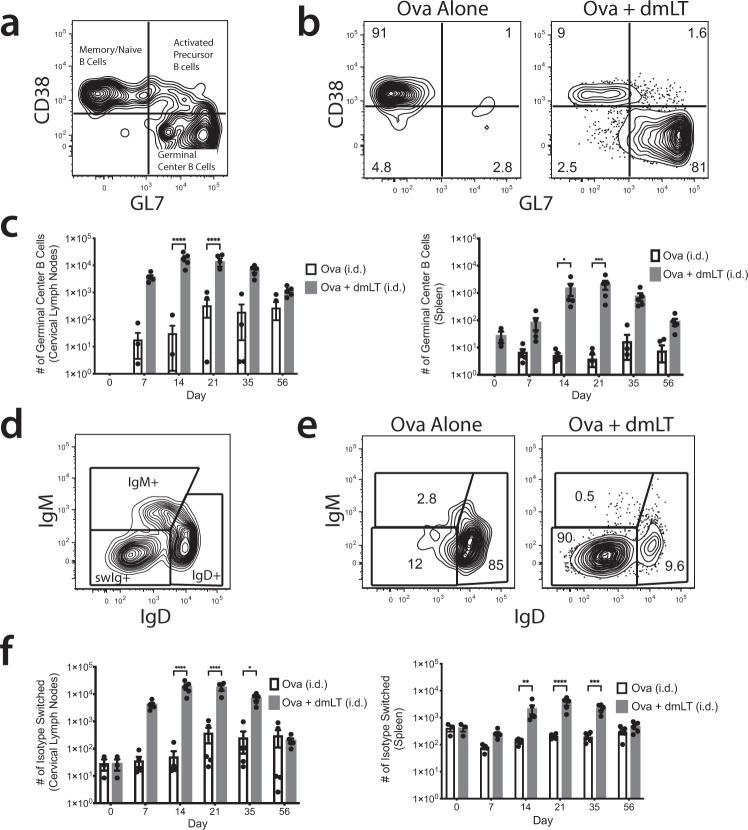
The peak of Ova-specific B cell activation occurs between 14 and 21 days post intradermal immunization. **a** Gating strategy for germinal center (GC) B cells. Ears of WT C57Bl/6 mice were intradermally injected with 10 μg of Ova or 10 μg of Ova plus 1 μg of dmLT. Mice were euthanized at the designated time points described in the methods. At each time point, cervical lymph nodes (CLNs) and the spleen were harvested and stained with decoy and tetramer. Throughout the figure, all tetramer positive Ova-specific B cells are displayed. **b** Representative flow plots of Ova-specific B cells at day 14 that expressed GC markers (CD38^−^, GL7^+^). **c** Number of Ova-specific B cells expressing the GC phenotype over the time course. **d** Gating strategy for isotype-switched (swIg) Ova-specific B cells. **e** Representative flow plots of Ova-specific B cells at day 14 that are swIg (IgM−, IgD−). **f** Number of Ova-specific B cells that are swIg over the time course. *p < 0.05; **p < 0.01; ***p < 0.001; ****p < 0.0001. Statistical analysis was performed using a two-way ANOVA with Sidak’s multiple comparison test. Zero values cannot be plotted on logarithmic graphs, and so sample dots do not appear for zero values, although they were considered for statistical analysis. The results shown are representative of two independent experiments, *N* = 5 per experiment. Graphs represent the mean at each time point ± the S.E.M.

**Fig. 3:**
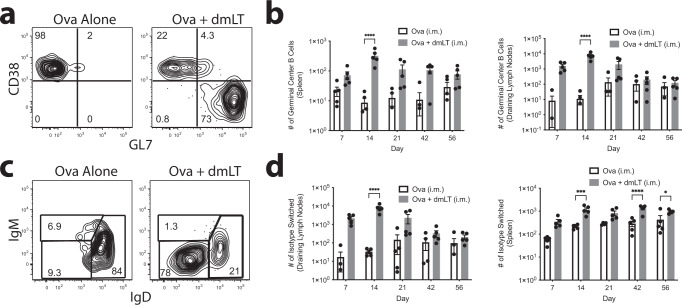
Intramuscular injection of dmLT induces peak of Ova-specific B cell activation at 14 days post-injection. Thighs of WT C57Bl/6 mice were intramuscularly injected with 10 μg of Ova or 10 μg of Ova plus 1 μg of dmLT. Mice were euthanized at the designated time points described in “Methods”. At each time point, inguinal lymph nodes, periaortic lymph nodes, popliteal lymph nodes (combined as the draining lymph nodes (DLNs)), and the spleen were harvested and stained with decoy and tetramer. **a** Representative flow plots of Ova-specific B cells at day 14 that expressed GC markers (CD38^−^, GL7^+^). **b** Number of Ova-specific B cells that are GC B cells over the time course. **c** Representative flow plots of Ova-specific B cells at day 14 that are swIg (IgM−, IgD−). **d** Number of Ova-specific B cells that are swIg over the time course. *p < 0.05; **p < 0.01; ***p < 0.001; ****p < 0.0001. Statistical analysis was performed using a two-way ANOVA with Sidak’s multiple comparison test. Zero values cannot be plotted on logarithmic graphs, and so sample dots do not appear for zero values, although they were considered for statistical analysis. The results shown are representative of two independent experiments, *N* = 5 per experiment. Graphs represent the mean at each time point ± the S.E.M.

A hallmark of T cell-dependent B cell activation and expansion is their entry into the germinal center (GC), where antibody class switch recombination and affinity maturation occur^33^. To assess whether B cells adopt the GC phenotype in our system, we used the surface markers CD38 and GL7 to define germinal center phenotype B cells (CD38^−^, GL7^+^) (Fig. 2a). When GC populations were examined over the 56-day time course following ID immunization, we found that Ova-specific GC B cells were elicited in the draining CLNs by day 7, peaking on days 14 and 21, and then plateauing by day 35 (Fig. 2b, c and Supplementary Fig. 1a). The spleen showed a slightly delayed response with GC B cells emerging on day 14, peaking on day 21, and returning to baseline by day 56 (Fig. 2c and Supplementary Fig. 1a). In mice receiving IM injections the draining lymph nodes showed a noticeable number of Ova-specific GC B cells by day 7, peaking on day 14, and decreasing for the rest of the time course (Fig. 3a, b and Supplementary Fig. 2a). There was a small but significant increase observed in the spleen on days 14, 21, and 35 (Fig. 3b).

We next assessed whether Ova-specific B cells experienced antibody class switch recombination based on their surface expression of IgD and IgM where isotype-switched B cells lose expression of IgD or IgM on the cell surface (Fig. 2d). When isotype switching was examined over the 56-day time course, we observed Ova-specific isotype-switched B cells in the draining CLNs appeared by day 7, peaked on day 14, and declined to a smaller but still detectable population by day 56 (Fig. 2e, f and Supplementary Fig. 1b). The spleen was similar to CLNs, but the peak was shifted to day 21 (Fig. 2f and Supplementary Fig. 1b). Mice receiving an IM injection displayed a nearly identical response in the draining lymph nodes to what was observed with ID injection (Fig. 3c, d and Supplementary Fig. 2b). The spleen had a lower but more persistent level of isotype switching (Fig. 3d and Supplementary Fig. 2b). Ultimately, vaccine-induced B cell activation is manifested in the secretion of vaccine-specific antibody (Ab). Next, we assessed how ID immunization affected vaccine-specific Ab production over time. Consistent with the delay between the expansion of Ova-specific B cells and isotype switching, anti-Ova IgG titers began to increase on day 21, just after the peak of the B cell response, and continued to increase until plateauing by day 35 (Supplementary Fig. 3). After IM immunization, Ab production closely mimicked that of ID injection at the early time points. (Supplementary Fig. 3). Interestingly, Abs did not level off with IM immunization but continued increasing until the end of the time course (Supplementary Fig. 3). Taken together, these data demonstrate that a single immunization with dmLT as the adjuvant induces a robust antigen (Ag)-specific B cell response that peaks 14 days following injection, regardless of route, although intriguingly, the antibody response to this single injection does not track exactly with the B cell peak where, at least for the IM injection, Ab levels continued to rise. Further, B cell adoption of the GC phenotype and subsequent isotype switching follow the increase in B cell numbers.

### A prime-boost injection with dmLT as the adjuvant enhances the Ova-specific B cell response

**Fig. 4:**
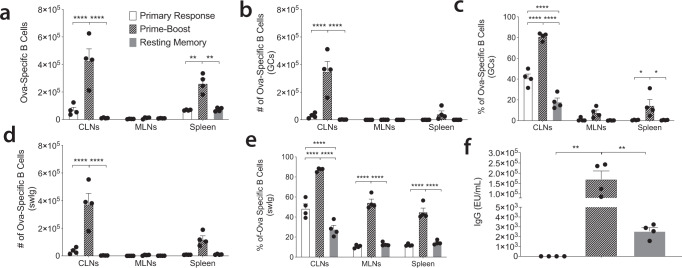
A booster injection of dmLT leads to an enhanced B cell response in distal lymph tissues. Ears of WT C57Bl/6 mice were intradermally injected with 10 μg of Ova plus 1 μg of dmLT. Resting memory tissue was collected 35 days after a single injection. Prime-boost mice received a prime injection and then a booster injection 28 days later. Seven days after the booster injection, the organs were harvested. Primary response tissue was collected 7 days after the single injection. Cervical lymph nodes (CLNs), mesenteric lymph nodes (MLNs), and the spleen were harvested and stained with decoy and tetramer. **a** Counts of the number of Ova-specific B cells; **b** and **c** are the number and percentage of GC B cells, respectively. **d**, **e** Number and percentage of isotype switched (swIg) B cells, respectively. **f** Plates were coated with Ova before the addition of serially diluted serum. IgG was detected using secondary antibodies conjugated to HRP against the IgG. *p < 0.05; **p < 0.01; ****p < 0.0001. Statistical analysis was performed using a two-way ANOVA with Sidak’s multiple comparison test. The results shown are representative of two independent experiments, *N* = 4. Graphs represent the mean in each organ + the S.E.M.

Most vaccines are delivered in a prime-boost fashion, sometimes with multiple boosts. This frequently results in superior B cell memory outcomes and higher affinity Ab following the boost leading to a more protective immune response. Although we initially observed that a single injection was sufficient to prime a vigorous B cell response, we posited that adding a booster injection would increase the response. To address this, we performed an ID injection in mice with Ova plus dmLT and then boosted the mice 28 days later. ID injection was chosen since it was equal to or better than IM immunization in the single injection experiments at eliciting B cell numbers. We then assessed the B cell response 1 week following the boost (35 days total) as previously described^34^. Here, we analyzed three groups: primary response (received a single injection on day 28 exemplifying a 1-week prime only), prime-boost (received an injection on day 0 and a second one on day 28, then assessed on day 35), and a group we dubbed resting memory (received a single injection on day 0 representing a 35-day prime only). Since previous work from our lab showed dmLT preferentially induced antigen-specific T cell migration to the mesenteric lymph nodes (MLNs) following ID injection, we investigated whether the same was true for B cells^13^. As expected, compared to the primary response and the resting memory, the prime-boost group had significantly higher numbers of Ova-specific B cells in the draining CLNs and spleen (Fig. 4a). While the increase of Ova-specific B cells in the CLNs is expected based on our previous data (Fig. 1a), the changes to the spleen highlight the effect of administering a booster injection^7^. Coinciding with overall B cell numbers, the prime-boost also increased the number of Ova-specific GC B cells and induced more isotype switching compared to either prime only or resting memory in the draining CLN but was not significant in the MLNs or spleen (Fig. 4b, d). While the overall numbers were not significantly different in all tissues, the percent of Ova-specific GC B cells in both CLN and spleen were significantly elevated in the prime-boost group (Fig. 4c). Further, the prime-boost group showed significant elevation in the percent of isotype-switched Ova-specific B cells in all tissues assessed (Fig. 4e). The increase in B cell activation in the prime-boost group was reflected in the serum antibody response. The prime-boost generated nearly 100-fold greater anti-Ova IgG compared to resting memory, while unsurprisingly, the prime only induced very little anti-Ova IgG (Fig. 4f). As expected, these data show that the benefits of using a prime-boost are to systemically enhance vaccine-specific B cell activation and that this translates to improved vaccine-specific antibody production.

### dmLT induces gut homing markers and migration of Ova-specific B cells to the intestine

One of the greatest barriers to developing effective vaccines against mucosal pathogens is that most parenterally delivered vaccines are unable to drive lymphocytes to enter mucosal tissues where they are most likely to be effective at combatting pathogens directly in those tissues^35,36^. We previously showed that a single ID injection with dmLT plus antigen induces antigen-specific CD4^+^ T cells to express the intestinal homing marker α_4_β_7_ and enhances migration to the lamina propria of the small and large intestines 14 days after injection^13^. Based on this, we hypothesized that dmLT would similarly induce this gut homing phenotype and migration in vaccine-specific B cells. Initially, we immunized mice once with dmLT plus Ova and assessed Ova-specific B cell migration into mucosal tissues. Unlike what we found for CD4 T cells, we observed that while dmLT induced a higher expression of α_4_β_7_ in Ova-specific B cells, these cells did not migrate into any mucosal tissue examined (Supplementary Fig. 4a–c). We adjusted our hypothesis to account for the possibility that a booster immunization would be essential for this type of B cell migration. Using the previously described prime-boost injection strategy (Fig. 4), we assessed whether dmLT imparted a mucosal homing phenotype on Ova-specific B cells. As we observed before using a single injection, mice that received a prime-boost immunization of Ova plus dmLT expressed the gut homing marker α_4_β_7_ on Ova-specific B cells in the draining CLNs, MLNs, and spleen as compared to Ova alone (Supplementary Fig. 4d)^37,38^.

**Fig. 5:**
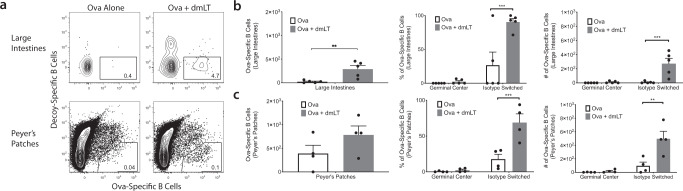
A booster injection of dmLT induces Ova-specific B cells to migrate to mucosal tissue. Ears of WT C57Bl/6 mice were intradermally injected with 10 μg of Ova or 10 μg of Ova plus 1 μg of dmLT. The mice received a booster injection 28 days later. Seven days after the booster injection, the organs were harvested. Lamina propria of the large intestines (LILP) and Peyer’s patches (PP) were harvested and stained with decoy and tetramer. **a** Representative flow plots of each of the tissues. **b** Counts of the number of Ova-specific B cells in the LILP and percentages and number of GC B cells and isotype switching. **c** Counts of the number of Ova-specific B cells in the PP and percentages and number of GC B cells and isotype switching. **p < 0.01; ***p < 0.001. Statistical analysis was performed using a Student’s *t*-test or two-way ANOVA with Sidak’s multiple comparison test. *N* = 2 or 3 per experiment, combined two independent experiments. Graphs represent the mean in each organ + the S.E.M.

While it was clear that dmLT could specifically induce the expression of intestinal homing markers on vaccine-specific B cells, the expression of these markers did not guarantee that the B cells entered mucosal tissues. Indeed, a single injection of dmLT plus antigen had no effect on mucosal homing. To ascertain whether B cells indeed migrate into the gut mucosa following a booster immunization, we isolated cells from the lamina propria of the small and large intestines and Peyer’s patches (PP) of the gut following a prime-boost immunization with Ova alone or Ova plus dmLT. We were able to identify a significant increase in total Ova-specific B cells in the tissues themselves in the dmLT group compared to the Ova alone group that was reflected in even greater isotype switching in the lamina propria of the large intestines (LILP) (Fig. 5a, b). While there was not a significantly greater number of Ova-specific B cells in the PP, dmLT induced significantly more isotype switching in the Ova-specific B cells that did appear in the PPs (Fig. 5a, c). Interestingly, we were unable to find any migration of Ova-specific B cells into the lamina propria of the small intestines.

### dmLT induces specific and non-circulating migration of isotype-switched Ova-specific B cells to multiple mucosal tissues

**Fig. 6:**
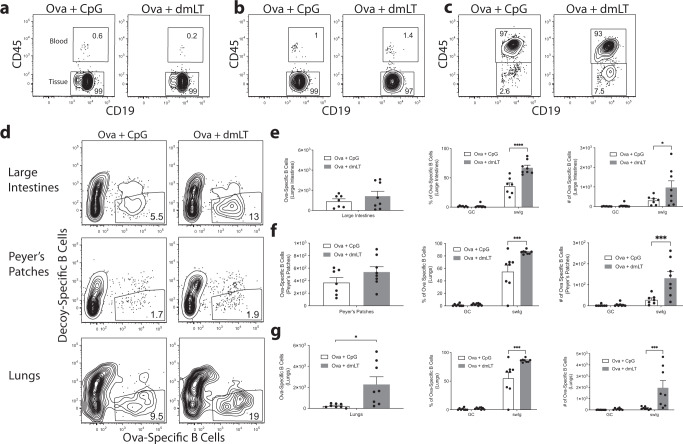
A booster injection of dmLT induces more Ova-specific B cells to migrate to mucosal tissues compared to CpG. Ears of WT C57Bl/6 mice were intradermally injected with 10 μg of Ova plus 1 μg of CpG or 10 μg of Ova plus 1 μg of dmLT. The mice received a booster injection 28 days later. Seven days after the booster injection, the organs were harvested. Following intravenous injection with anti-CD45 Ab (intravascular staining) to discriminate tissue-resident B cells from circulating cells, lamina propria of the large intestines (LILP), Peyer’s patches (PP), and cellular isolates of the lungs were harvested and stained with decoy and tetramer. Representative flow plots of anti-CD45 intravascular staining for **a** LILP, **b** PP, and **c** lungs. **d** Representative flow plots of each tissue. **e** The number of Ova-specific B cells in the LILP and percentages and number of GC B cells and isotype switching. **f** The number of Ova-specific B cells in the PP and percentages and number of GC B cells and isotype switching. **g** The number of Ova-specific B cells in the lungs and percentages and number of GC B cells and isotype switching. *p < 0.05; **p < 0.01; ***p < 0.001. Statistical analysis was performed using a Student’s *t*-test or two-way ANOVA with Sidak’s multiple comparison test. *N* = 3–5 per experiment, combined two independent experiments. Graphs represent the mean in each organ + the S.E.M.

It was possible that the gut homing phenotype we observed was universally attributable to B cell activation and was not associated with the use of the dmLT adjuvant. CpG is a toll-like receptor 9 agonist that can be administered intradermally and is currently used in approved human vaccines^39^. To assess whether the gut homing phenotype was unique to dmLT, we compared a prime-boost regimen using either CpG or dmLT. While the total number of Ova-specific B cells was similar between the two groups in all lymphoid tissues indicating that there was no difference in the overall capacity for each adjuvant to induce B cells Supplementary Fig. 5a), there was significantly greater expression of α_4_β_7_ in the dmLT immunized mice in CLNs and MLNs compared to mice immunized with CpG (Supplementary Fig. 5b). To determine if these antigen-specific B cells preferentially entered the gut mucosa, we repeated this experiment and measured the presence of Ova-specific B cells in the LILP and PP. To demonstrate that these cells were non-circulating B cells and thus were most likely resident cells, we injected mice intravenously with fluorochrome-labeled anti-CD45 Ab 3 min prior to euthanasia to mark all hematopoietic cells in the blood at the time when the mice were euthanized. This delineated circulating from tissue-resident cells, as others have described^40–42^. Notably, nearly all the B cells we found in the intestinal tissues were not labeled with anti-CD45 and therefore were non-circulating, implying they were resident to the tissue examined (Fig. 6a, b). When we assessed only tissue-resident B cells, we determined that both CpG and dmLT were able to induce Ova-specific B cells that potentially trafficked to the LILP and PPs although dmLT induced more of these cells in each tissue (Fig. 6d–f); however, mice that received dmLT had a significantly higher quantity of antigen-specific isotype-switched B cells compared to the mice injected with CpG (Fig. 6e, f). This suggested that parenteral dmLT acts as a more potent activator of the B cells that adopt intestinal residence. Additionally, few of the Ova-specific B cells expressed the GC phenotype indicating that the B cells were likely activated or reactivated at a distal site and then migrated to the intestines, although since we did not explore GC kinetics at the mucosal sites, we cannot rule out the possibility that B cells in mucosal sites were activated there in response to antigen draining from the injection site.

The recent pandemic outbreak of the respiratory virus SARS-CoV-2 is affecting millions of people globally. Based on the fact the mice immunized ID with dmLT-adjuvanted vaccines are better protected against pulmonary infection with *Pseudomonas aeruginosa*^14^, we hypothesized that dmLT might be capable of specifically inducing vaccine-specific B cell migration into the lungs of mice. To test this, we performed the prime-boost experiment described above for gut homing but analyzed Ova-specific B cells in the lungs. Although it was clear that many of the cells in the lungs were in the blood, as expected, it was also apparent that there was a significant population of cells that resided in the lungs and did not stain with intravascular Ab (Fig. 6c). Notably, there were significantly more non-circulating, antigen-specific B cells in the lungs following prime-boost injection of dmLT compared to CpG (Fig. 6d, g). Like the intestines, dmLT promoted more significant isotype switching (Fig. 6g). As before, there was a lack of Ova-specific GC B cells, suggesting that these B cells are potentially migrating into the lungs after being activated elsewhere.

### Intradermally administered dmLT is superior to mucosally administered dmLT or to CpG by any route at inducing mucosal humoral immunity

**Fig. 7:**
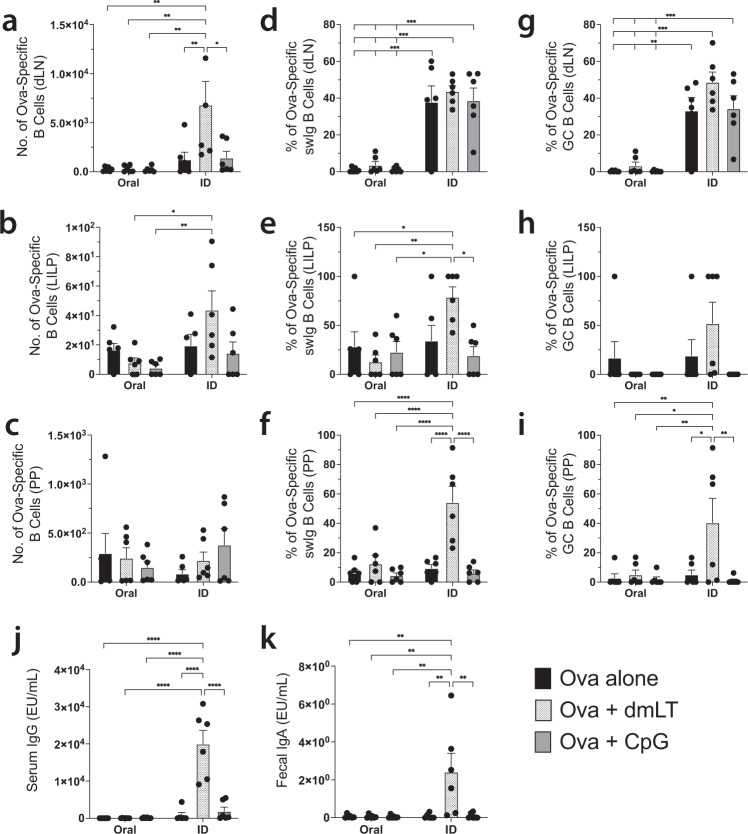
Intradermal immunization with dmLT is more potent than oral immunization at the same dose. WT C57Bl/6 mice were intradermally injected or orally gavaged with 10 μg of Ova alone, 10 μg of Ova plus 1 μg of CpG, or 10 μg of Ova plus 1 μg of dmLT. The mice received a booster immunization 28 days later by the same route as the initial immunization for each group. Seven days after the boost, organs were harvested. Following intravascular staining with anti-CD45 to discriminate tissue-resident B cells from circulating cells, draining lymph nodes (dLN), lamina propria of the large intestines (LILP), and Peyer’s patches (PP) were harvested and stained with decoy and tetramer. **a**–**c** Counts of the number of Ova-specific B cells, **d**–**f** percentage isotype-switched (swIg) Ova-specific B cells and **g**–**i** percentage germinal center (GC) phenotype Ova-specific B cells are compared. Ova-specific **j** serum IgG and **k** fecal secreted IgA were assessed by ELISA for each group. *p < 0.05; **p < 0.01; ***p < 0.001; ***p < 0.0001. Statistical analysis was performed using a two-way ANOVA with Tukey’s multiple comparisons test. *N* = 3 per experiment, combined two independent experiments. Graphs represent the mean by each route + the S.E.M.

The conventional method for inducing mucosal immunity is via mucosal vaccine delivery. We next sought to test how ID administration of dmLT would fare against a mucosal immunization and how this would compare to CpG, also administered by both routes. Mice were prime-boost immunized as previously described with Ova plus either dmLT or CpG by either the oral or ID route. We chose to immunize orally due to the substantial body of literature that dmLT can act as an adjuvant in both mice and humans via the oral route^43–47^. To make the fairest determination of how ID immunization might compare to mucosal immunization, we chose to utilize the same dose of antigen and adjuvant for both routes. We found that ID-administered dmLT was superior at inducing total Ova-specific B cell numbers in injection-site draining lymph nodes compared to antigen alone or antigen plus CpG (Fig. 7a), although the percentage of isotype-switched or germinal center B cells was not different between the ID groups (Fig. 7b, c). Oral administration induced very little to no response regardless of the adjuvant used. The same held true when we assessed the total numbers of Ova-specific B cells in the LILP where ID dmLT was superior to CpG or oral administration (Fig. 7b). Total numbers of Ova-specific B cells in the PPs did not differ significantly between any of the groups (Fig. 7c); however, isotype switching in both the LILP and the PPs was significantly induced by ID dmLT immunization compared to CpG or oral administration with either adjuvant (Fig. 7e, f). GC phenotype cells were not significantly different in the LILP between groups (Fig. 7h) but were once again higher in the ID dmLT group in the PPs (Fig. 7i). While B cell responses and migration to mucosal sites were superior using ID administration, we sought to determine how systemic antibodies might be affected. To do this, we measured serum IgG and IgA in each immunization group. While we observed no appreciable serum IgA with no differences between groups, we did find that Ova-specific serum IgG was highest in the ID-administered dmLT group compared to all other treatments (Fig. 7j). A hallmark of mucosal humoral immunity is the production of secreted IgA at the mucosal immunization site^48,49^. We next assessed the production of fecal IgA responses in immunized mice where the oral administration might be expected to induce a robust response. Instead, we found that ID dmLT administration produced significantly more Ova-specific fecal IgA compared to any other group (Fig. 7k). This demonstrates that, at the same vaccine dose, ID-delivered dmLT is superior to oral dmLT or CpG administered by either route for inducing both antigen-specific B cells as well as systemic and mucosal antibody responses.

### Ova-specific B cell germinal center phenotype, isotype switching, and Peyer’s Patch migration are dependent on the transcription factor Batf3

**Fig. 8:**
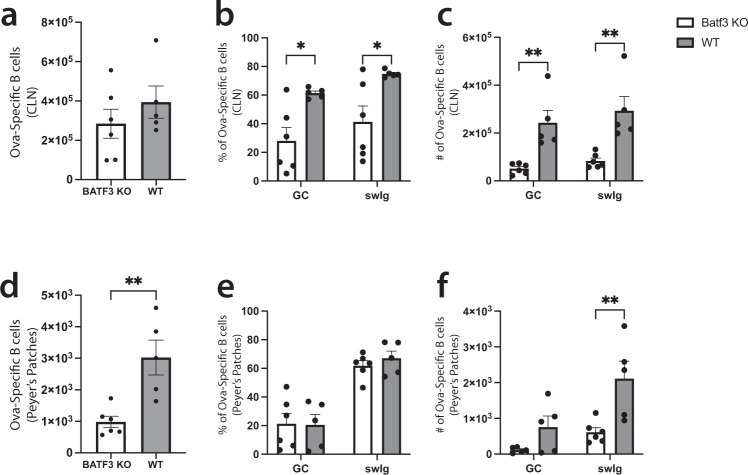
Batf3 is required for dmLT-induced Ova-specific B cell germinal center phenotype, isotype switching, and migration to Peyer’s Patches. Ears of WT or Batf3 KO mice were intradermally injected with 10 μg of Ova plus 1 μg of dmLT. Mice received a booster injection 28 days later. Seven days after the booster injection, the organs were harvested. Following intravascular staining to discriminate tissue-resident B cells from circulating cells, draining cervical lymph nodes (CLN) and Peyer’s Patches were harvested and stained with decoy and tetramer. The total number of **a**, **d** Ova-specific B cells, **b**, **e** percentages, and **c**, **f** number of GC B cells and isotype-switched cells are shown for **a**–**c** CLN and **d**–**f** Peyer’s Patches. *p < 0.05; **p < 0.01. Statistical analysis was performed using a **a**, **d** Student’s *t*-test or **b**, **c**, **e**, **f** two-way ANOVA with Sidak’s multiple comparison test. *N* = 5–6, representing two independent experiments. Graphs represent the mean in each strain ± the S.E.M.

Our previous work demonstrated that dmLT-induced antigen-specific CD4 T cell migration into the gut is partly dependent on the presence of the basic leucine zipper transcription factor ATF-like 3 (Batf3)^13^; however, it is unclear whether Batf3 has a similar effect on vaccine-specific B cell migration to mucosal tissues. To test this, we immunized WT mice or Batf3^−/−^ mice with dmLT and Ova as before in a prime-boost fashion and assessed Ova-specific B cell migration into mucosal tissues as well as GC formation and isotype switching. While the lack of Batf3 had no significant effect on the number of Ova-specific B cells in the injection-site draining CLN (Fig. 8a), both GC formation and isotype switching were severely compromised (Fig. 8b, c). Notably, Ova-specific B cell migration into the lungs and LILP was not affected by the lack of Batf3 (Supplementary Fig. 6); however, migration of these cells into the PP was significantly reduced in Batf3^−/−^ mice (Fig. 8d). While the percentage of GC phenotype and isotype-switched Ova-specific B cells was similar in the PPs, the total numbers for each were significantly reduced (Fig. 8e, f). Some studies have shown that Batf3 has potential impacts on B cell activation, migration, and isotype switching that is potentially mediated by loss of conventional dendritic cells or effects on T follicular helper cells^50–54^. We hypothesized that these Batf3 deficient extrinsic factors might be playing a role in the observed defective B cell response. To test this, we transferred bulk CD45.1^+^ congenic, purified wild-type B cells into either CD45.2^+^ expressing wild-type mice or Batf3^−/−^ mice and immunized each group with dmLT plus Ova as previously described. We postulated that the transferred cells would lose the capacity for isotype switching and PP migration due to altered signaling in the Batf3^−/−^ recipient compared to the wild-type recipient. Instead, we found that there was no defect in the Ova-specific transferred cells to either migrate to the PPs or to undergo isotype switching or germinal center formation in the Batf3^−/−^ recipient mice compared to the wild-type recipient mice (Supplementary Fig. 7). Taken together, these data show that a non-mucosal injection of dmLT can induce isotype-switched antigen-specific B cells that migrate to, and establish residence in, multiple mucosal tissues and that, at least for PPs, this migration is dependent on the non-extrinsic presence of the transcription factor Batf3.

## Discussion

Undoubtedly, vaccines are one of the greatest medical interventions for the betterment of human health ever devised. While nearly all current vaccines contain an adjuvant, the addition of novel adjuvants to a wide variety of vaccines has increased their potency and generated a revolution in vaccine design; however, it is often unclear exactly how adjuvants work in the generation of a vaccine-induced immune response or how they might be used to manipulate immunity to favor protection against certain pathogens, particularly those that invade via the mucosa. Notably, as we have previously demonstrated, it is possible to utilize adjuvants, specifically dmLT, to target intestinal tissues for residence by vaccine-specific CD4 T cells, but it is not at all clear whether adjuvants can impart a similar migratory phenotype on vaccine-specific B cells which would allow them to occupy local mucosal tissues where they may have the capacity to elicit infection neutralizing antibodies locally^13^. Here we sought to assess how dmLT might regulate mucosal B cell immunity when administered parenterally.

Our results represent an important and previously unappreciated link between parenteral immunizations and humoral mucosal immunity. We demonstrate that intradermal injections, using dmLT as the adjuvant, increase the number of antigen-specific B cells in the intestinal and pulmonary tissues as well as draining lymph nodes. Importantly, these cells are not circulating in the blood but are, in fact, in the mucosal tissues themselves. In the intestines, nearly all these cells are resident in both the lamina propria of the large intestines and the Peyer’s Patches. While many of the B cells in the lung are in the blood, as others have shown, a sizable portion of these cells are in the lung tissue itself^18,40^. Previous work has demonstrated that CD4 and CD8 T cells in mucosal tissues have distinct phenotypes from those T cells found in the blood and lymph nodes^55^. Similar work has been done with B cells, demonstrating that blood and intestinal B cells are heterogeneous populations and are distinctly different from one another^19,56^. One strategy developed to get these lymphocytes to mucosal tissues is the “prime and pull” method, where a prime injection is given parenterally, and the boost is given at the desired mucosal surface to pull the primed memory lymphocytes into that mucosal tissue^57^. This approach has proven successful at getting CD4 and CD8 T cells into the vagina and the lungs and provides protection against HSV-2 and tuberculosis, respectively^57–60^. However, “prime and pull” does not seem to be as effective for B cells as has been assessed after intramuscular immunization against Hepatitis B antigen with CpG/alum as the adjuvant or against the HIV antigen gp140 with MPL-A or CCL28 as the adjuvant^12,61^. We were able to find antigen-specific, isotype-switched B cells in the lungs, Peyer’s patches, and lamina propria of the large intestines after two intradermal immunizations using dmLT as the adjuvant and forgoing the pull booster, suggesting that dmLT can act as a potent mucosal B cell directing adjuvant that does not require the boost to be administered mucosally but rather exclusively parenterally. Notably, we compared the intradermal route to immunizing via a mucosal route by giving dmLT and Ova orally. We specifically chose to compare vaccination by these two routes using the same dose of adjuvant and antigen to make the fairest comparison of immune induction in the gut tissue. There are a multitude of studies showing that the intradermal route is “dose sparing,” which allows the use of a lower dose by the intradermal route compared to other routes^62–64^. We anticipated that the intradermal route would be at least comparable to oral immunization but were surprised to find that the intradermal route was superior to the oral route. This was true at the dose we used for both routes and pertained to both B cell migration to gut tissues as well as secretory gut IgA responses. This further highlights the “dose-sparing” capacity of the intradermal route. It has been known for over a century that the oral route may be tolerogenic to administered antigens but that this can be overcome with certain adjuvants^65–68^. Indeed, dmLT has been successfully used in mice and humans as oral adjuvants, and so this was the logical route for use to choose with this adjuvant as a mucosal comparison to the intradermal route^43–47^. It is possible that increasing the dose of oral dmLT could equal immune responses that we observed with intradermal immunization, although we propose that this would circumvent the benefits of intradermal immunization, such as allowing for the use of much lower doses capable of inducing pan-mucosal humoral immunity. Considering this, incorporating dmLT into current and developing vaccines could greatly enhance their mucosal response after intradermal injection and increase their efficacy against pathogens that enter via mucosal sites.

Our research also addresses an important gap in the current knowledge of vaccination and how altering parenteral vaccine composition might regulate mucosal immunity. It is known that administering intradermal or intramuscular vaccines with certain adjuvants can induce a secretory IgA response in some mucosal tissues; however, little is known about how these types of vaccinations target B cell migration into those same mucosal tissues^2,69,70^. Experiments from over 40 years ago showed that oral exposure to antigen causes B cells in the Peyer’s patches to undergo isotype switching and migrate to the lamina propria^71,72^. The data presented here demonstrate for the first time, to our knowledge, that antigen-specific B cells can potentially travel from the draining lymph nodes to mucosal tissues following immunization in a distant, non-mucosal site. Since our data show that an intradermal administration of an antigen with dmLT can drive antigen-specific B cells to the Peyer’s patches and lamina propria of the large intestines, dmLT could help improve the efficacy of vaccines against enteric pathogens such as *Shigella* or enterotoxigenic *Escherichia coli*. Additionally, our finding that B cells migrate into the lung after intradermal immunization shows the potential for using dmLT to protect against a wide variety of mucosal pathogens, including respiratory pathogens such as influenza or SARS-CoV-2. This study adds to the body of literature showing that certain non-mucosal vaccines can induce mucosal immune responses. For example, it has been shown that Venezuelan equine encephalitis virus replicon particles given in the footpads of mice can induce IgA against co-administered antigens in multiple mucosal sites^73^.

Interestingly, and akin to our previous findings showing that Batf3 plays a role in CD4 T cell migration to the intestines, we found that Batf3 likewise played a role in vaccine-mediated B cell migration to Peyer’s Patches of the small intestine^13^. We also demonstrated that both germinal center phenotype and isotype switching was hampered in vaccine-specific B cells from Batf3^−/−^ mice in response to intradermal dmLT immunization. Some studies have already implicated Batf3 in regulating isotype switching, likely through the influence of CD103^+^ dendritic cells^50–53^; however, most of these studies assessed isotype switching in mucosal tissues in response to mucosal challenge or vaccination; thus, little is known regarding how Batf3 effects isotype switching following parenteral immunization, as we show here. To our knowledge, this is the first demonstration that Batf3 is important for vaccine-specific B cell migration into gut-associated lymphoid tissues. One possibility is that there is a specific defect in migration as the number of Ova-specific B cells in the injection-site draining lymph nodes did not differ significantly between WT and Batf3^−/−^ mice. Another possibility is that migrating cells are unable to survive in the Peyer’s Patches and die upon entry. Interestingly, the lack of Batf3 on all non-B cells, where the deficiency was extrinsic to the B cells, had no effect on B cell migration to Peyer’s patches or isotype switching and germinal center phenotype formation. This potentially contrasts with other studies where dendritic cell Batf3 expression is important for these B cell functions. We are further investigating these possibilities to better understand and optimize parenteral vaccine-mediated mucosal immunity.

Additionally, our study demonstrated that while an initial priming immunization was excellent at generating a potent and expanded vaccine-specific B cell response, it was incapable of inducing mucosal migration. This finding implies that memory B cells must be recalled with a booster response to initiate bona fide mucosal homing and residence. It is possible that our experimental approach was unable to detect a very small number of B cells that had mucosally migrated after the initial priming event; however, we and others have shown that the magnetic bead enrichment approach for both T and B cells is exceptional at identifying small numbers of antigen-specific lymphocytes^74–78^. This prime-boost requirement has implications for why these types of vaccines induce such robust protection against mucosal pathogens. For example, the newest mRNA-based vaccines against the SARS-CoV-2 virus are only around 50% effective following the initial priming but increase to 95% effectiveness with a booster vaccination^79^. Our findings imply that the booster immunization directly induces IgA-producing lung or gut resident B cell population, although this remains to be explored and is a limitation of the current study. Additionally, we did not assess whether other mucosal tissues, such as the female reproductive tract, are targeted by dmLT adjuvanticity. A final limitation of our study is whether these mucosal B cells are required for protection against pathogen challenges. Future work will assess whether the vaccine-established mucosal B cells (and T cells) contribute to protection against mucosal pathogens such as influenza in the airways or *Salmonella* in the gut. The ability of dmLT to confer this mucosal homing property has the potential to aid future vaccine development as more than half of children under 2 years of age receive the *Haemophilus influenzae* B conjugate vaccine or Pneumococcal conjugate vaccine^80^. One possibility for exploiting the mucosal homing properties of dmLT would be to conjugate dmLT directly to the polysaccharide antigens of various pathogens, thereby providing simultaneous adjuvanticity, mucosal homing, and B cell help in a single vaccine. Based on our study, this would have the benefit of targeting isotype-switched, memory cellular, and humoral immunity to the mucosal tissues. Our study is the first to demonstrate how a parenteral immunization can drive vaccine-specific B cells into mucosal tissues and adds to the body of literature demonstrating that adjuvant choice can drastically regulate vaccine-mediated immunity, particularly at portals of pathogen entry.

## Methods

### Study design

This study sought to measure the effect that dmLT has on antigen-specific B cells. Mice were intradermally or intramuscularly injected with Ovalbumin alone, Ovalbumin and dmLT, or Ovalbumin and CpG. The number of antigen-specific B cells and percentage of germinal center and isotype switching were used to determine the activation of B cells. α_4_β_7_ and intravascular staining were used to determine the migration and residence of antigen-specific B cells. IgG ELISAs were used to elucidate the quantity of antigen-specific antibodies. The size of experiment groups is specified in the figure legends. All analyses were conducted unblinded.

### Ethics statement

This study was carried out in accordance with recommendations from the Guide for the Care and Use of Laboratory Animals of the National Institutes of Health. Tulane University is accredited by the Association for Assessment and Accreditation of Laboratory Animal Care (AAALAC). All experimental procedures involving animals were approved and performed in compliance with the guidelines established by Tulane University School of Medicine’s Institutional Animal Care and Use Committee.

### Mice and immunizations

C57BL/6J, B6.SJL-Ptprc^a^ Pepc^b^/BoyJ (CD45.1 congenic), and BATF3^−/−^ mice were purchased from Jackson Laboratory (Bar Harbor, ME) and were maintained under specific-pathogen-free conditions in the vivarium at Tulane University School of Medicine. BATF3^−/−^ mice were bred in-house and used for experimentation at approximately 8 weeks of age. Mice were injected with 10 μg of Ovalbumin, 10 μg of Ovalbumin with 1 μg dmLT, or 10 μg of Ovalbumin with 1 μg CpG at 6–10 weeks of age either intradermally in both ears or intramuscularly in both thighs. Boost injections were given 4 weeks post the initial injection. For oral immunizations, mice were given the same doses as above of each adjuvant and antigen in 100 μl PBS using an oral gavage needle to deliver the vaccine intragastrically. At designated times post-infection, mice were euthanized by CO_2_ asphyxiation, and cervical lymph nodes (CLNs), mesenteric lymph nodes (MLNs), spleens, secondary lymph nodes (popliteal, inguinal, and paraaortic lymph nodes), Peyer’s patches, and large intestines were harvested for flow cytometry. Serum was collected via cardiac puncture for ELISAs.

### Creation of Ova and decoy tetramers

We followed the protocol established in ref. ^81^. To do this, ovalbumin was biotinylated at a 1:1 ratio of biotin to protein using the commercially available EZ-link Sulfo-NHS-LC-Biotinylation kit (Thermo). Next, streptavidin, already conjugated to phycoerythrin (SA-PE), was added at a 6:1 ratio of Ovalbumin to SA-PE to create the final tetramer. Decoy tetramer was produced in a similar manner, with an irrelevant biotinylated peptide (EAWGALANWAVDSA—called 2W1S) used in place of Ovalbumin to allow for the detection, and exclusion, of B cells that bound to the non-Ovalbumin tetramer components (SA/biotin/PE). For the decoy tetramer, biotinylation of the peptide was carried out as above (1:1 ratio of biotin to peptide using the EZ-link Sulfo-NHS-LC-Biotinylation kit). DyLight650 was also included as a fluorophore to distinguish it from the Ovalbumin-containing tetramer.

### Generation of single-cell preparations, B cell tetramer staining, magnetic assisted cell sorting, and flow cytometry

CLNS, MLNs, DLNs, PPs, and spleens were made in single-cell suspensions by homogenizing the organs over a 100-μm nylon mesh filter in cold sorter buffer (1× phosphate-buffered saline, 2% newborn calf serum, and 0.1% sodium azide). For lung cell isolation, the lungs were collected and minced in IMDM media (MilliporeSigma) supplemented with 1× penicillin-streptomycin, 1× glutamine (Mediatech), and 10% heat-inactivated FBS (Invitrogen), followed by incubation for 60 min with tissue culture grade type IV collagenase (1 mg/ml; MiiliporeSigma) in a 37 °C orbital shaker at 100 rpm. The cell suspension was then filtered through a sterile 70-μm nylon filter to create single-cell preparations. Lamina propria from the large intestine (LILP) was isolated^82^. To do this, cleaned LILP was cut into 1 cm pieces and incubated in a 5 mM ethylenediaminetetraacetic acid (EDTA) and 5 mM dithiothreitol (DTT) solution in Hank’s balanced salt solution (HBSS) at 37 °C and shook at 220 rpm for 15 min. The tissue was then washed over a 100-μm filter and incubated again at 37 °C, and shaken at 220 rpm in a 5 mM EDTA and HBSS solution for 20 min. After washing over a 100-μm filter, the tissue was further cut into smaller pieces. It was then transferred into a digestions buffer (HBSS with calcium and magnesium, 10% FBS, 0.2 U/ml of Liberase (Sigma), and 200 U/ml DNase 1 (Sigma)) and at 37 °C and shaken at 220 rpm for 30 min. The solution was filtered through a 70-μm filter before continuing with single-cell preparations that were resuspended in 50 μl of FcBlock with 1 μM PE-Cy5 decoy for 5 min at room temperature, and then 1 μM of Ova-PE B cell tetramer was added for 25 min on ice in the dark, and the cells were washed. Next, the cells were stained with anti-PE magnetic beads (Miltenyi) and passed over an LS column on a quadroMACS magnet. Eluted cells were stained with the following anti-mouse antibodies at a 1:100 dilution for each: Anti-GL7 eFluor 450 (Invitrogen Cat: 48-5902-82 Clone: GL-7), Anti-CD3e BV510 (Biolegend Cat: 100353 Clone: 145-2C11), Anti-CD11c BV510 (Biolegend Cat: 117338 Clone N418), Anti-F4/80 BV510 (Biolegend Cat: 123135 Clone: BM8), Anti-IgD FITC (Biolegend Cat: 405704 Clone: 11-26 c.2a), Anti-CD19 PE-Cy7 (Biolegend Cat: 115520 Clone: 6D5), Anti-IgM APC (BD Cat: 550676 Clone II/41), Anti-CD38 AF700 (Invitrogen Cat: 56-0381-82 Clone: 90), Anti-α4β7 BV421 (BD Cat: 747758 Clone DATK32), Anti-IgD BV605 (BD Cat: 563003 Clone: 11-26 c.2a), Anti-CCR9 FITC (Biolegend Cat: 128706 Clone: CW-1.2), Anti-GL7 PerCP-Cy5.5 (Biolegend Cat: 144609 Clone: GL-7), Anti-IgD BV510 (BD Cat: 563110 Clone: 11-26 c.2a), Anti-CD45.1 FITC (eBioscience Cat: 11-0453-85 Clone A20), Anti-CD3e PerCP-Cy5.5 (Tonbo Cat: 65-0031-U100 Clone 145-2C11), Anti-CD11c PerCP-Cy5.5 (eBioscience Cat: 45-0114-82 Clone N418), Anti-F4/80 PerCp-Cy5.5 (Tonbo Cat: 65-4801-U100 Clone BM8.1), Anti-CD45.2 APC-Cy7 (Tonbo Cat: 25-0454-U100 Clone: 104). To identify circulating cells, mice were injected with 2 μg of Anti-CD45 BV605 (Biolegend Cat: 103140 Clone: 30-F11) retro-orbitally 3 min before euthanasia^40–42^. Cells were collected on an LSRFortessa (Beckton-Dickson). Data were analyzed using FlowJo software (TreeStar, Ashland, OR).

### B cell adoptive transfer

Spleens from CD45.1 congenic mice were made into single-cell suspensions by homogenizing the organs over a 100-μm nylon mesh filter in cold PBS under sterile conditions, followed by RBC lysis. B cells were then purified according to the EasySep (StemCell Technologies) Mouse B Cell Isolation Kit instructions. B cells were then magnetically enriched and counted. B cell purity was approximately 95%, and 3 × 10^7^ total B cells were transferred into each mouse via retroorbital injection.

### Antibody ELISAs

Serum total Ig antibody ELISAs were performed using a 96-well round-bottom (Greiner Bio-one). The wells of the plates were coated with 1 μg/ml of Ova or an external recombinant mouse IgG (Invitrogen) or IgA (SouthernBiotech) standard overnight at 4 °C. Plates were blocked with 2% BSA prior to an overnight incubation with serially diluted samples sample incubation at 4 °C. The samples were detected using horseradish peroxidase-conjugated anti-mouse IgG (Thermo Fisher) or IgA (SouthernBiotech). The absorbance was read at 450 nm, and the data were analyzed using a sigmoidal dose-response with least-squares fit. The results were quantitated as the number of ELISA units (EU) per milliliter using the average for two sample dilutions closest to the midpoint of the standard curve. For fecal IgA measurements, fecal pellets were freshly collected, weighed, and PBS with a P8340 protease inhibitor cocktail (Sigma-Aldrich) was added at a ratio of 1 ml PBS to 0.1 g mouse feces to equalize the liquid volume across fecal samples. Feces was then homogenized and centrifuged at 16,000 × *g* for 10 min, and supernatants were stored at −80 °C until assessed for anti-Ova IgA as described above.

### Purification of dmLT protein

dmLT was produced from *E. coli* clones expressing recombinant protein derived from the human ETEC isolate *E. coli* H10407 as others have described^43,83,84^. To do this, *E. coli* bacteria were lysed using a microfluidizer. The resulting lysed product was then dialyzed and fractionated using D-galactose affinity chromatography. Eluted, purified protein was passed through high-capacity endotoxin removal resin (Thermo Fisher Scientific) and was lyophilized and stored at 4 °C until use. Vials of lyophilized dmLT were freshly resuspended in ultrapure H_2_O at a concentration of 1 mg/ml prior to use.

### Statistical analysis

Statistical differences between data sets were assessed using ordinary one-way analysis of variance (ANOVA) with Tukey’s multiple comparison test or two-way ANOVA with Tukey’s or Sidak’s multiple comparison test (GraphPad Prism software). Outliers were removed using the Grubbs outlier test. Significant differences were noted as *p < 0.05; **p < 0.01; ***p < 0.001; ****p < 0.0001.

### Reporting summary

Further information on research design is available in the Nature Research Reporting Summary linked to this article.

## Supplementary information

Supplementary Figures for NPJVACCINES-02291R1

REPORTING SUMMARY

## Data Availability

All data are available in the main text or the supplementary materials.
