# Supplementary material for: Establishment of isotype-switched, antigen-specific B cells in multiple mucosal tissues using non-mucosal immunization
Source: NPJ Vaccines. 2023 May 31;8:80. doi: 10.1038/s41541-023-00677-z (PMC10231862; doi:10.1038/s41541-023-00677-z)
Supplement: Supplementary file 1 — Supplementary Figures for NPJVACCINES-02291R1 [file 41541_2023_677_MOESM1_ESM.pdf]

Supplemental Figures

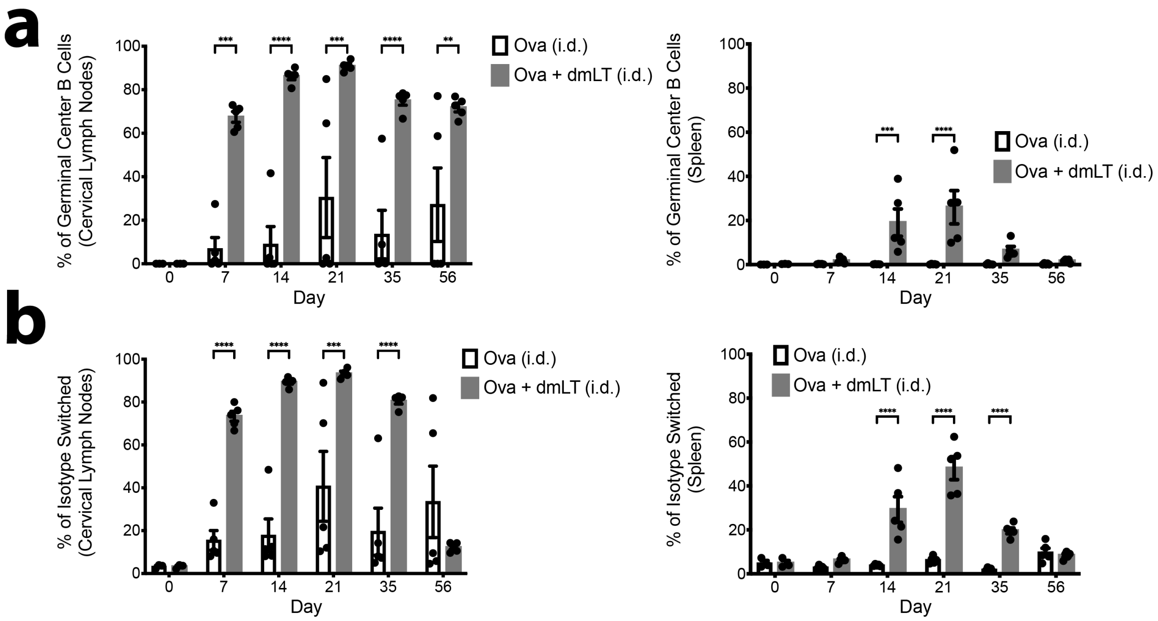

Supplemental Figure 1. Peak activation of Ova-specific B cell happens at 14 to 21 days post intradermal injection. Ears of WT C57Bl/6 mice were intradermally injected with 10μg of Ova or 10μg of Ova plus 1μg of dmLT. Mice were euthanized at the designated time points described in the methods. At each time point, cervical lymph nodes (CLNs) and the spleen were harvested and stained with decoy and tetramer. **a** Changes, as a percentage of Ova-specific B cells, of GC B cells over the time course. **b** Changes, as a percentage of Ova-specific B cells, of swIg over the time course. **, p<0.01; ***, p<0.001; ****, p <0.0001. Statistical analysis was performed using a two-way ANOVA with Sidak's multiple comparison test. Results shown are representative of two-independent experiments, N=5. Graphs represent the mean at each timepoint ± the S.E.M.

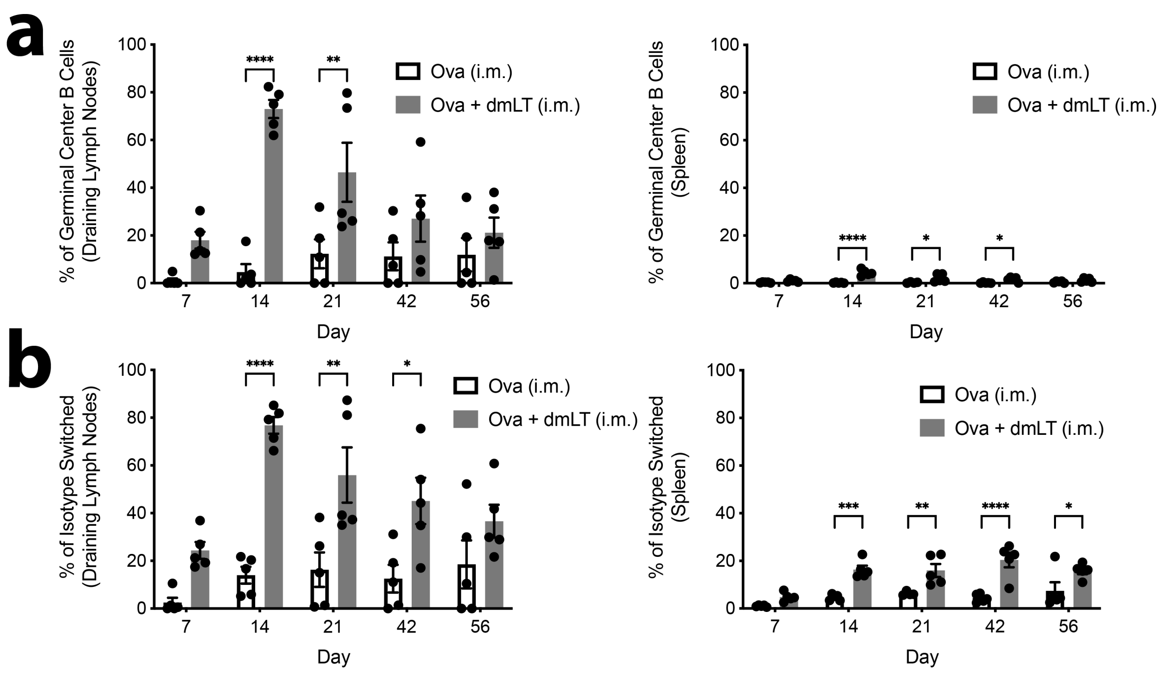

Supplemental Figure 2. Intramuscular injection of dmLT induces peak of Ova-specific B cell activation at 14 days post injection. Thighs of WT C57Bl/6 mice were intramuscularly injected with 10µg of Ova or 10µg of Ova plus 1µg of dmLT. Mice were euthanized at the designated time points described in the methods. At each time point, inguinal lymph nodes, periaortic lymph nodes, popliteal lymph nodes (combined as the draining lymph nodes (DLNs)) and the spleen were harvested and stained with decoy and tetramer. **a** Changes, as a percentage of Ova-specific B cells, of GC B cells over the time course. **b** Changes, as a percentage of Ova-specific B cells, of swIg over the time course. *, $p<0.05$; **, $p<0.01$; ***, $p<0.001$; ****, $p <0.0001$. Statistical analysis was performed using a two-way ANOVA with Sidak's multiple comparison test. Results shown are representative of two-independent experiments, N=5. Graphs represent the mean at each timepoint ± the S.E.M.

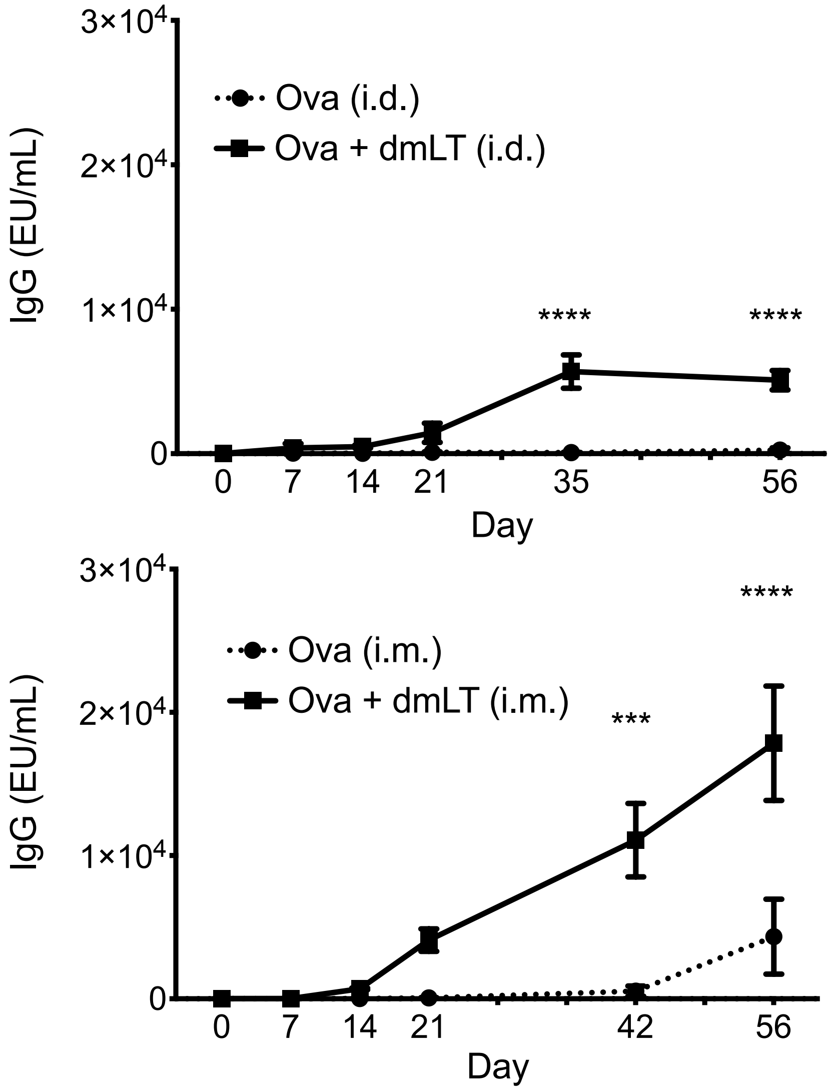

Supplemental Figure 3. Ova specific IgG increases after 21 days post injection. (Top) Ears of WT C57Bl/6 mice were intradermally injected with 10μg of Ova or 10μg of Ova plus 1μg of dmLT. Mice were euthanized at the designated time points described in the methods. At each time point, serum was collected and stored at -80°C until ready to use. (Bottom) Thighs of WT C57Bl/6 mice were intramuscularly injected with 10μg of Ova or 10μg of Ova plus 1μg of dmLT. Mice were euthanized at the designated time points described in the methods. At each time point, serum was collected and stored at -80°C until ready to use. Plates were coated with Ova before the addition of serially

diluted serum. IgG was detected using secondary antibodies conjugated to HRP against the IgG. ***, $p<0.001$; ****, $p <0.0001$. Statistical analysis was performed using a two-way ANOVA with Sidak's multiple comparison test. Results shown are representative of two-independent experiments, N=5. Graphs represent the mean at each timepoint ± the S.E.M.

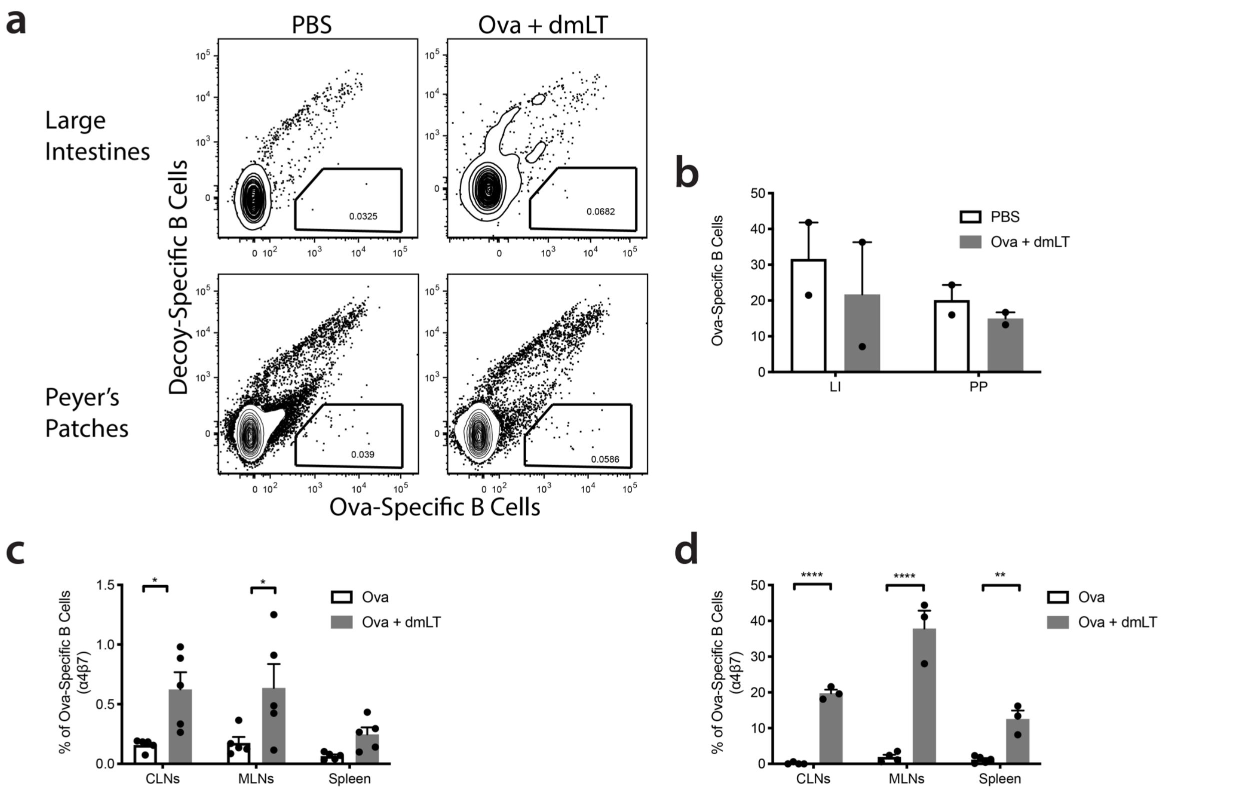

Supplemental Figure 4. A single injection of dmLT induces $\alpha_4\beta_7$ expression but not migration to mucosal tissues. **a** and **b** Ears of WT C57Bl/6 were intradermally injected with PBS or 10µg of Ova plus 1µg of dmLT. 14 days later, lamina propria of the large intestines (LI) and Peyer's patches were harvested and stained with decoy and tetramer. **b** Representative flow plots of Ova-specific B cells and B) the number of Ova-specific B cells. C and D) Ears of WT C57Bl/6 were intradermally injected with 10µg of Ova or 10µg of Ova plus 1µg of dmLT. **c** 14 days later, cervical lymph nodes (CLNs), mesenteric lymph nodes (MLNs), and the spleen were harvested and stained with decoy and tetramer. Percentage of $\alpha_4\beta_7$+ Ova-specific B cells are compared. **d** The mice received then a boost injection 28 days later. 7 days after the boost injection, the organs were harvested. CLNs, mesenteric lymph nodes MLNs, and the spleen were harvested and stained with decoy and tetramer. Percentage of $\alpha_4\beta_7$+ Ova-specific B

cells are compared. *, $p<0.05$; **, $p<0.01$; ***, $p<0.001$; ****, $p <0.0001$. Statistical analysis was performed using a two-way ANOVA with Sidak's multiple comparison test. N=2-5, 2 independent experiments. Graphs represent the mean in each organ + the S.E.M.

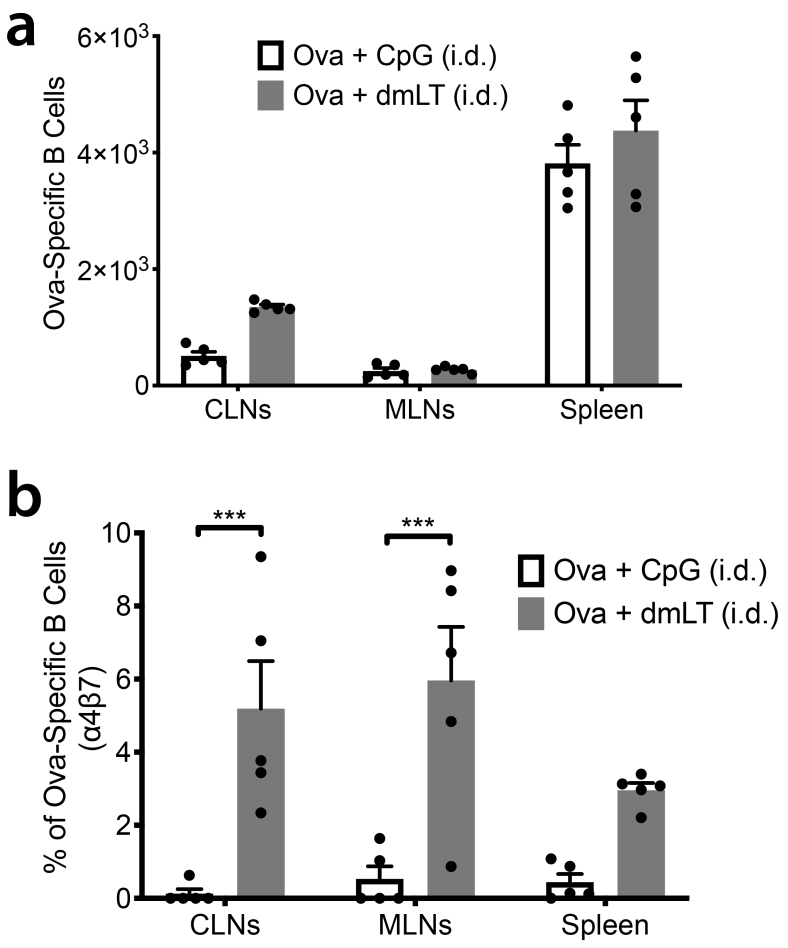

Supplemental Figure 5. dmLT is able to increase the expression of α4β7 on Ova-specific B cells compared to CpG. Ears of WT C57Bl/6 mice were intradermally injected with 10μg of Ova plus 1μg of CpG or 10μg of Ova plus 1μg of dmLT. 14 days after a single injection the cervical lymph nodes (CLNs), mesenteric lymph nodes (MLNs), and the spleen were harvested and stained with decoy and tetramer. **a** Counts of the number of Ova-specific B cells and **b** percentage α4β7 expression are compared. ***, $p<0.001$. Statistical analysis was performed using a two-way ANOVA with Sidak's multiple comparison test. Results shown are representative of two-independent experiments, N=5. Graphs represent the mean in each organ + the S.E.M.

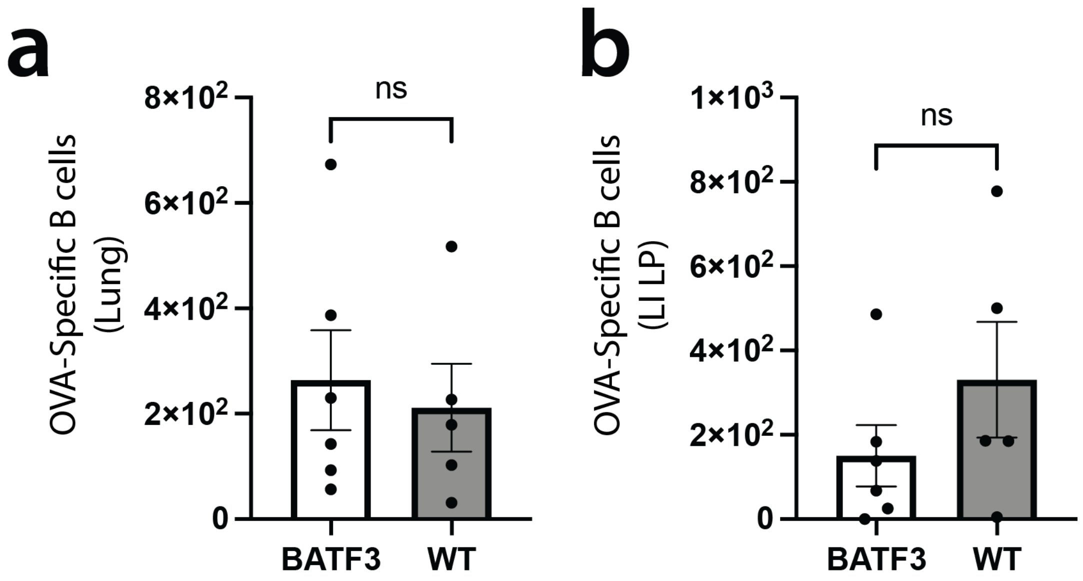

Supplemental Figure 6. Batf3 is not required for dmLT-induced Ova-specific B cell migration to lungs or LILP. Ears of WT or Batf3 KO mice were intradermally injected with 10μg of Ova plus 1μg of dmLT. Mice received a boost injection 28 days later. 7 days after the boost injection, the organs were harvested. Following intravascular staining to discriminate tissue resident B cells from circulating cells, lungs and LILP were harvested and stained with decoy and tetramer. The total number of Ova-specific B cells are shown for **a** Lung and **b** LILP. ns, not significant; Statistical analysis was performed using a Student's t test. N=5-6, representing 2 independent experiments. Graphs represent the mean in each strain ± the S.E.M.

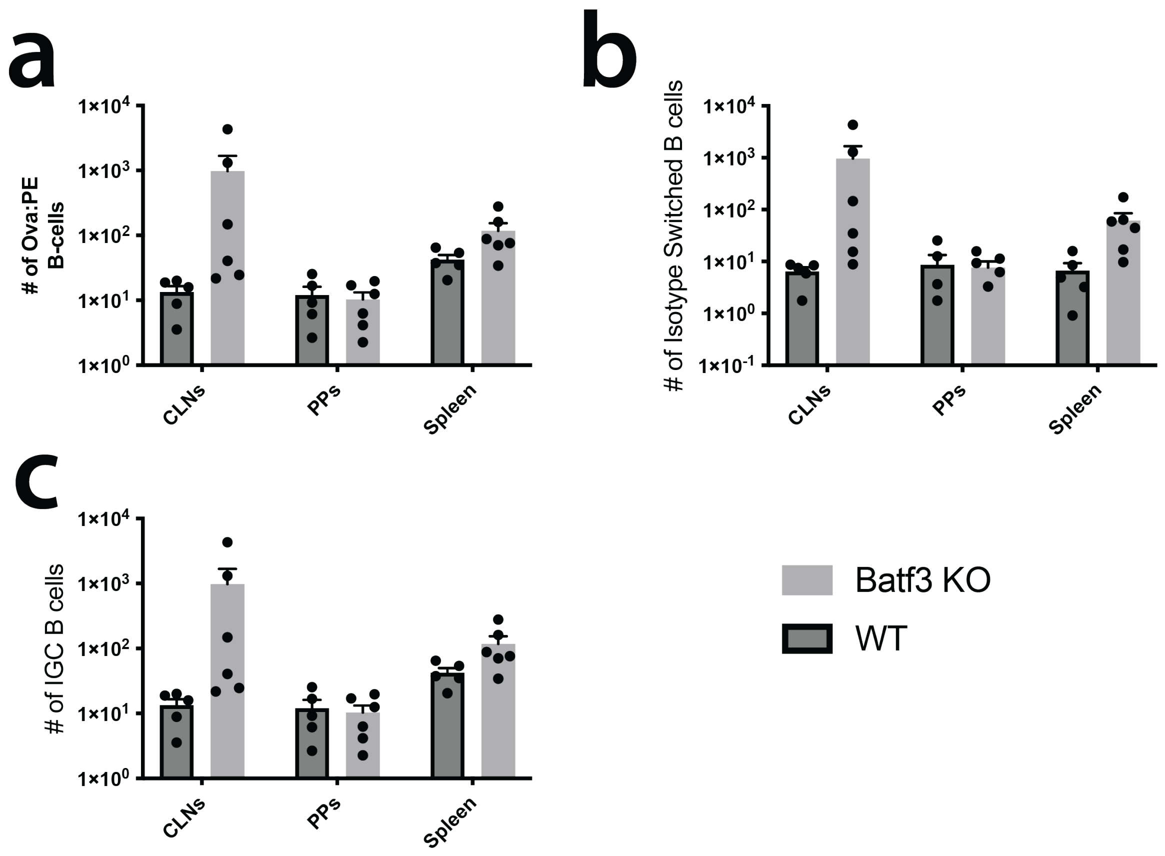

Supplemental Figure 7. Extrinsic Batf3 does not significantly affect dmLT-induced Ova-specific B cell migration or activation. WT CD45.1^{+} B cells were purified, and 3 x 10^{7} B cells were injected intravenously into CD45.2 WT or Batf3$^{-/-}$ mice. Mice with transferred cells were intradermally injected with 10µg of Ova plus 1µg of dmLT. Mice received a boost injection 28 days later. 7 days after the boost injection, organs were harvested. Following intravascular staining to discriminate tissue resident B cells from circulating cells, Ova-specific B cells were assessed in the draining CLN, PP, and spleens. **a** total number, **b** isotype switched, and **c** germinal center Ova-specific B cells are shown for each organ; Statistical analysis was performed using a two-way ANOVA with Sidak's multiple comparison test with no significant difference found between the

two groups in any organ. N=5-6, representing 2 independent experiments. Graphs represent the mean in each organ + the S.E.M.
