# Supplementary material for: Establishment of isotype-switched, antigen-specific B cells in multiple mucosal tissues using non-mucosal immunization
Source: NPJ Vaccines. 2023 May 31;8:80. doi: 10.1038/s41541-023-00677-z (PMC10231862; doi:10.1038/s41541-023-00677-z)
Supplement: Supplementary file 2 — REPORTING SUMMARY [file 41541_2023_677_MOESM2_ESM.pdf]

nature portfolio

Corresponding author(s): Dr James McLachlan

Last updated by author(s): May 10, 2023

Reporting Summary

Nature Portfolio wishes to improve the reproducibility of the work that we publish. This form provides structure for consistency and transparency in reporting. For further information on Nature Portfolio policies, see our Editorial Policies and the Editorial Policy Checklist.

Statistics

For all statistical analyses, confirm that the following items are present in the figure legend, table legend, main text, or Methods section.

n/a	Confirmed	
☐	☒	The exact sample size (*n*) for each experimental group/condition, given as a discrete number and unit of measurement
☐	☒	A statement on whether measurements were taken from distinct samples or whether the same sample was measured repeatedly
☐	☒	The statistical test(s) used AND whether they are one- or two-sided<br>*Only common tests should be described solely by name; describe more complex techniques in the Methods section.*
☐	☒	A description of all covariates tested
☐	☒	A description of any assumptions or corrections, such as tests of normality and adjustment for multiple comparisons
☐	☒	A full description of the statistical parameters including central tendency (e.g. means) or other basic estimates (e.g. regression coefficient) AND variation (e.g. standard deviation) or associated estimates of uncertainty (e.g. confidence intervals)
☐	☒	For null hypothesis testing, the test statistic (e.g. *F*, *t*, *r*) with confidence intervals, effect sizes, degrees of freedom and *P* value noted<br>*Give P values as exact values whenever suitable.*
☒	☐	For Bayesian analysis, information on the choice of priors and Markov chain Monte Carlo settings
☒	☐	For hierarchical and complex designs, identification of the appropriate level for tests and full reporting of outcomes
☒	☐	Estimates of effect sizes (e.g. Cohen's *d*, Pearson's *r*), indicating how they were calculated

Our web collection on statistics for biologists contains articles on many of the points above.

Software and code

Policy information about availability of computer code

Data collection	BD FACSDiva Software Version 9.0.1
Data analysis	GraphPad Prism Version 9.5.1

For manuscripts utilizing custom algorithms or software that are central to the research but not yet described in published literature, software must be made available to editors and reviewers. We strongly encourage code deposition in a community repository (e.g. GitHub). See the Nature Portfolio guidelines for submitting code & software for further information.

Data

Policy information about availability of data

All manuscripts must include a data availability statement. This statement should provide the following information, where applicable:

- Accession codes, unique identifiers, or web links for publicly available datasets
- A description of any restrictions on data availability
- For clinical datasets or third party data, please ensure that the statement adheres to our policy

Data collected in this study are presented in the paper

Human research participants

Policy information about studies involving human research participants and Sex and Gender in Research.

Reporting on sex and gender	n/a
Population characteristics	n/a
Recruitment	n/a
Ethics oversight	n/a

Note that full information on the approval of the study protocol must also be provided in the manuscript.

Field-specific reporting

Please select the one below that is the best fit for your research. If you are not sure, read the appropriate sections before making your selection.

☒ Life sciences ☐ Behavioural & social sciences ☐ Ecological, evolutionary & environmental sciences

For a reference copy of the document with all sections, see nature.com/documents/nr-reporting-summary-flat.pdf

Life sciences study design

All studies must disclose on these points even when the disclosure is negative.

Sample size	The sample size was determined using previously published experiments in the same field
Data exclusions	No data was excluded
Replication	All experiments had at least two replicates and were consistent between the experiments
Randomization	Mice were chosen at random to determine which group they were part of
Blinding	Blinding was not done as the results were discrete measured events

Reporting for specific materials, systems and methods

We require information from authors about some types of materials, experimental systems and methods used in many studies. Here, indicate whether each material, system or method listed is relevant to your study. If you are not sure if a list item applies to your research, read the appropriate section before selecting a response.

Materials & experimental systems

n/a	Involved in the study	
☐	☒	Antibodies
☒	☐	Eukaryotic cell lines
☒	☐	Palaeontology and archaeology
☐	☒	Animals and other organisms
☒	☐	Clinical data
☒	☐	Dual use research of concern

Methods

n/a	Involved in the study	
☒	☐	ChIP-seq
☐	☒	Flow cytometry
☒	☐	MRI-based neuroimaging

Antibodies

Antibodies used	Anti-GL7 eFluor 450 Invitrogen Cat: 48-5902-82 Clone: GL-7, Anti-CD3e BV510 Biolegend Cat: 100353 Clone: 145-2C11, Anti-CD11c BV510 Biolegend Cat: 117338 Clone N418, Anti-F4/80 BV510 Biolegend Cat: 123135 Clone: BM8, Anti-IgD FITC Biolegend Cat: 405704 Clone: 11-26c.2a, Anti-CD19 PE-Cy7 Biolegend Cat: 115520 Clone: 6D5, Anti-IgM APC BD Cat: 550676, Anti-CD38 AF700 Invitrogen Cat: 56-0381-82 Clone: 90, Anti-a4B7 BV421 BD Cat: 747758, Anti-IgD BV605 BD Cat: 563003, Anti-CCR9 FITC Biolegend Cat: 128706 Clone: CW-1.2, Anti-GL7 PerCP-Cy5.5 Biolegend Cat: 144609, Anti-CD45 BV605 Biolegend Cat: 103140 Clone: 30-F11, Anti-IgD BV510 BD Cat: 563110, Anti-CD45.1 FITC eBioscience Cat: 11-0453-85 Clone A20, Anti-CD3e PerCP-Cy5.5 Tonbo Cat: 65-0031-U100, Anti-CD11c PerCP-Cy5.5 eBioscience Cat: 45-0114-82 Clone N418, Anti-F4/80 PerCp-Cy5.5 Tonbo Cat: 65-4801-U100, Anti-CD45.2 APC-Cy7 Tonbo Cat: 25-0454-U100 Clone: 104

Validation	All antibodies were used in accordance to manufacturers guidlines

Animals and other research organisms

Policy information about studies involving animals; ARRIVE guidelines recommended for reporting animal research, and Sex and Gender in Research

Laboratory animals	6-8 week old male and female C57Bl/6, B6.SJL-Ptprca Pepcb/BoyJ, and BATF3 KO mice were used
Wild animals	n/a
Reporting on sex	Sex differences were compared and there was no difference between male and female mice
Field-collected samples	*For laboratory work with field-collected samples, describe all relevant parameters such as housing, maintenance, temperature, photoperiod and end-of-experiment protocol OR state that the study did not involve samples collected from the field.*
Ethics oversight	IACUC at Tulane University approved all experiments involving mice

Note that full information on the approval of the study protocol must also be provided in the manuscript.

Flow Cytometry

Plots

Confirm that:

☐ The axis labels state the marker and fluorochrome used (e.g. CD4-FITC).

☒ The axis scales are clearly visible. Include numbers along axes only for bottom left plot of group (a 'group' is an analysis of identical markers).

☒ All plots are contour plots with outliers or pseudocolor plots.

☒ A numerical value for number of cells or percentage (with statistics) is provided.

Methodology

Sample preparation	CLNS, MLNs, DLNs, PPs, and spleens were made in single cell suspensions by homogenizing the organs over a 100 µm nylon mesh filter in cold sorter buffer (1x phosphate buffered saline, 2% newborn calf serum, and 0.1% sodium azide). For lung cell isolation, the lungs were collected and minced in IMDM media (MilliporeSigma) supplemented with 1× penicillin-streptomycin, 1x glutamine (Mediatech), and 10% heat-inactivated FBS (Invitrogen), followed by incubation for 60 minutes with tissue culture grade type IV collagenase (1 mg/mL; MiiliporeSigma) in a 37°C orbital shaker at 100 rpm.LI was cut into 1cm pieces and incubated in a 5mM ethylenediaminetetraacetic acid (EDTA) and 5mM dithiothreitol (DTT) solution in Hank's balanced salt solution (HBSS) at 37oC and shook at 220rpm for 15 minutes. The tissue was then washed over a 100 µM filter and incubated again at 37oC and shook at 220rpm in a 5mM EDTA and HBSS solution for 20 minutes. After washing over a 100 µM filter, the tissue was further cut into smaller pieces. It was then transferred into a digestions buffer (HBSS with calcium and magnesium, 10% FBS, 0.2 U/mL of Liberase (Sigma), and 200 U/mL DNase 1 (Sigma)) and at 37oC and shook at 220rpm for 30 minutes.
Instrument	BD Fortessa LRII
Software	Diva was used to collect Flow Cytometry data and FlowJo was used to analyze the data
Cell population abundance	No sorting was performed
Gating strategy	The lymphocyte population was determined using SSC-A vs. FSC-A, singlets were determined using SSC-A vs. SSC-W, B cells were determined by lineage negative cells (CD11c, CD3, and F4/80) vs. CD19, Tetramer positive B cells were gated off of the B cell positive population and determined by Decoy vs. Tetramer, germinal centers were determined from the Tetramer positive population and determined by CD38 vs. GL7, antibody isotypes were determined from the Tetramer positive population and determined by IgM vs. IgD, alpha4-beta7 positive B cells were determined from the Tetramer positive population and determined by alpha4-beta7 vs. FSC-A. Resident B cells were determined after the B cell population was determined and determined by CD45 vs. CD19.

☒ Tick this box to confirm that a figure exemplifying the gating strategy is provided in the Supplementary Information.
